# Extracellular volume fraction determined by equilibrium contrast-enhanced computed tomography: correlation with histopathological findings in gastric cancer

**DOI:** 10.1007/s11604-023-01393-3

**Published:** 2023-02-03

**Authors:** Yusuke Nishimuta, Daisuke Tsurumaru, Satohiro Kai, Junki Maehara, Yoshiki Asayama, Eiji Oki, Kousei Ishigami

**Affiliations:** 1 Department of Clinical Radiology, Graduate School of Medical Sciences, Kyushu University, 3-1-1 Maidashi, Higashi-Ku, Fukuoka, 812-8582 Japan; 2 Department of Endoscopic Diagnostics and Therapeutics, Kyushu University Hospital, 3-1-1 Maidashi, Higashi-Ku, Fukuoka, 812-8582 Japan; 3 Department of Anatomic Pathology, Graduate School of Medical Sciences, Kyushu University, 3-1-1 Maidashi, Higashi-Ku, Fukuoka, 812-8582 Japan; 4 Department of Radiology, Faculty of Medicine, Oita University, Yufu City, Oita, 879-5593 Japan; 5 Department of Surgery and Science, Graduate School of Medical Sciences, Kyushu University, 3-1-1 Maidashi, Higashi-Ku, Fukuoka, 812-8582 Japan

**Keywords:** Gastric cancer, Computed tomography, Extracellular volume fraction

## Abstract

**Purpose:**

To assess the relationship between histopathological features of gastric cancer and the extracellular volume fraction (ECV) measured by preoperative equilibrium contrast-enhanced computed tomography (CECT).

**Materials and methods:**

The study group consisted of 66 patients with surgically resected gastric adenocarcinoma who underwent preoperative multiphasic CECT. Tumor ECVs were calculated using region-of-interest measurements within the gastric cancer and aorta of each case on unenhanced and equilibrium-phase images. The relationship between the mean ECV values and clinicopathological parameters was examined by univariate analysis. Parameters showing a significant difference in the former test were further tested by linear regression and receiver operating characteristic (ROC) curve analyses.

**Results:**

In the univariate analysis, the values of venous invasion (*p* = 0.0487) and tumor infiltration (INF) pattern (*p* < 0.0001) were significantly correlated with the tumor ECV. INF was significantly correlated (*β* = 0.57, *p* < 0.0001) in the linear regression analysis. The tumor ECV showed better diagnostic accuracy for predicting INF (INFa/b vs INFc), and the area under the ROC curve value was 0.89.

**Conclusion:**

Tumor ECV determined by equilibrium CECT is significantly correlated with the pathological INF of gastric cancer.

## Introduction

Gastric cancer (GC), the fourth leading cause of cancer-related death, is a malignant neoplasm that continues to have a poor prognosis worldwide [1]. Histopathological factors, including the depth of tumor invasion (T stage), histological type, differentiation, lymphovascular invasion, infiltration pattern, and lymph node metastasis, have a close relationship with the aggressiveness of the disease, treatment strategies, and patient prognosis [2–6]. Recent advances in histochemical and molecular biological techniques have made it possible to identify different prognostic factors of GCs [7–9]. Pathological and molecular prognostic biomarkers of GC are generally examined in tissue samples obtained from surgery and endoscopic biopsy. However, tissue sampling is invasive, costly, and prone to undersampling for unresectable tumors, and it is, therefore, impractical for long-term ongoing patient monitoring. A simple non-invasive pre-therapeutic biomarker that can be easily repeated over time would be highly beneficial for the management of patients with GC.

Computed tomography (CT) is a standard imaging modality for the pre-therapeutic staging of GC and is mainly used to determine the depth of tumor invasion and the presence/absence of nodal or distant metastases [10–12]. Several authors have reported the contrast-enhanced CT (CECT) features of GC associated with histopathological characteristics. Takao et al. [13] reported that GC composed of marked fibrous tissue stroma showed gradual enhancement on triphasic spiral CT and revealed that the entire tumor was depicted most clearly in the equilibrium phase. Tsurumaru et al. [14] reported that undifferentiated-type GCs showed peak enhancement with significantly higher attenuation values than other types of GC in the delayed phase. They also reported that most of the early GCs with ulceration composed of peritumoral fibrosis demonstrated weak enhancement in the portal phase, with gradual enhancement on the delayed phase on triphasic contrast-enhanced CT [15]. These results suggested that histological fibrosis is one of the important factors affecting the CECT enhancement pattern of GCs.

The extracellular volume fraction (ECV) is the sum of the extravascular extracellular volume fraction and the intravascular space fraction, which represents the total stromal space. The ECV determined by equilibrium CECT or magnetic resonance (MR) imaging was shown to be associated with the pathological fibrosis volume, and the ECV has been well validated for the evaluation of cardiac and hepatic fibrosis [16–19]. Several research groups recently reported the use and utility of ECV in oncological assessments. Benjaminsen et al. [20] observed that the ECV fraction in human melanoma xenografts measured by contrast-enhanced MR imaging was positively correlated with the extravascular extracellular volume fraction determined by histological analysis. Adams et al. [21] found that MR T1 mapping with ECV measurement could represent a novel in vivo biomarker for the classification of renal cell carcinomas according to their nucleolar grade. Fukukura et al. [22] reported that the tumor ECV determined by equilibrium CECT was a useful imaging biomarker to predict the survival of pancreatic ductal adenocarcinoma patients after chemotherapy.

To the best of our knowledge, the clinical relevance of the ECV measured by equilibrium CECT as an imaging biomarker for patients with GC has not been reported. We speculated that the ECV fraction of tumor tissue quantified by equilibrium CECT that can be easily integrated into routine examinations would be an important imaging biomarker for patients with GC. We conducted this initial exploratory study to evaluate the correlation between the ECV of primary tumors and clinicopathological factors of GC.

## Materials and methods

This was a retrospective analysis of routine examination of GC cases at a single university hospital. Ethics committee approval was granted by the local institutional ethics review board, with a waiver of written informed consent.

### Patients

The cases of 106 consecutive patients with pathologically proven gastric adenocarcinoma who underwent CT scanning prior to surgical resection during the 4-year period from January 2012 to December 2015 were collected for this study. The patients were retrospectively identified through a review of our CT database and records at the Department of Radiology. The exclusion criteria were as follows: (a) neoadjuvant chemotherapy before surgery (*n* = 6); (b) no contrast or poor contrast (*n* = 17); (c) poor visualization of the tumor due to insufficient distention of the stomach (*n* = 14); and (d) previous distal gastrectomy (*n* = 3). In total, the cases of 66 patients (33 men, 33 women; mean age, 65.5 years; range, 32–86 years) who had undergone a CT examination prior to the resection of GC were analyzed. Data pertaining to the following clinical and biochemical variables were retrieved: age, sex, hematocrit level, and serum levels of tumor marker carbohydrate antigen (CA) 19–9 and carcinoembryonic antigen (CEA), which were measured within 1 week of the CT examination.

### CT image acquisition

All patients signed informed consent for the CECT examination. After an overnight fast, each patient ingested 5.25 g of an effervescent agent (Baros Effervescent Granules-S; Horii Pharmaceutical Industries, Saitama, Japan) with a small amount of water just before the scanning to achieve gastric pouch distension. All patients underwent scanning with a 320-detector row CT (Aquilion ONE; Canon Medical Systems, Tokyo). After unenhanced CT images were acquired, multiphasic CECT scans were performed with a bolus-triggered technique: 2 mL/kg non-ionic contrast material (Iopamiron 370; Bayer Health Care, Osaka, Japan) was injected for a fixed duration of 30 s at a variable injection rate by an automated power injector. The monitoring frequency from 10 s after the contrast injection was 1/s. The trigger threshold was an increase of100 Hounsfield units (HU) in the descending aorta. The delay from the trigger to the initiation of the scan was 15 s. The portal venous and equilibrium phases were acquired at 60 and 240 s, respectively. The scan parameters were as follows: rotation time, 0.5 s; section thickness and intervals, 0.5 mm; and 120 kVp, 200 mAs, and 512 × 512 matrix. The scanning covered the entire stomach during a single breath-hold [14]. All CT datasets were transferred to a commercially available workstation equipped with image reconstruction software (Synapse Vincent, Fujifilm, Tokyo).

### Image analysis

**Fig. 1:**
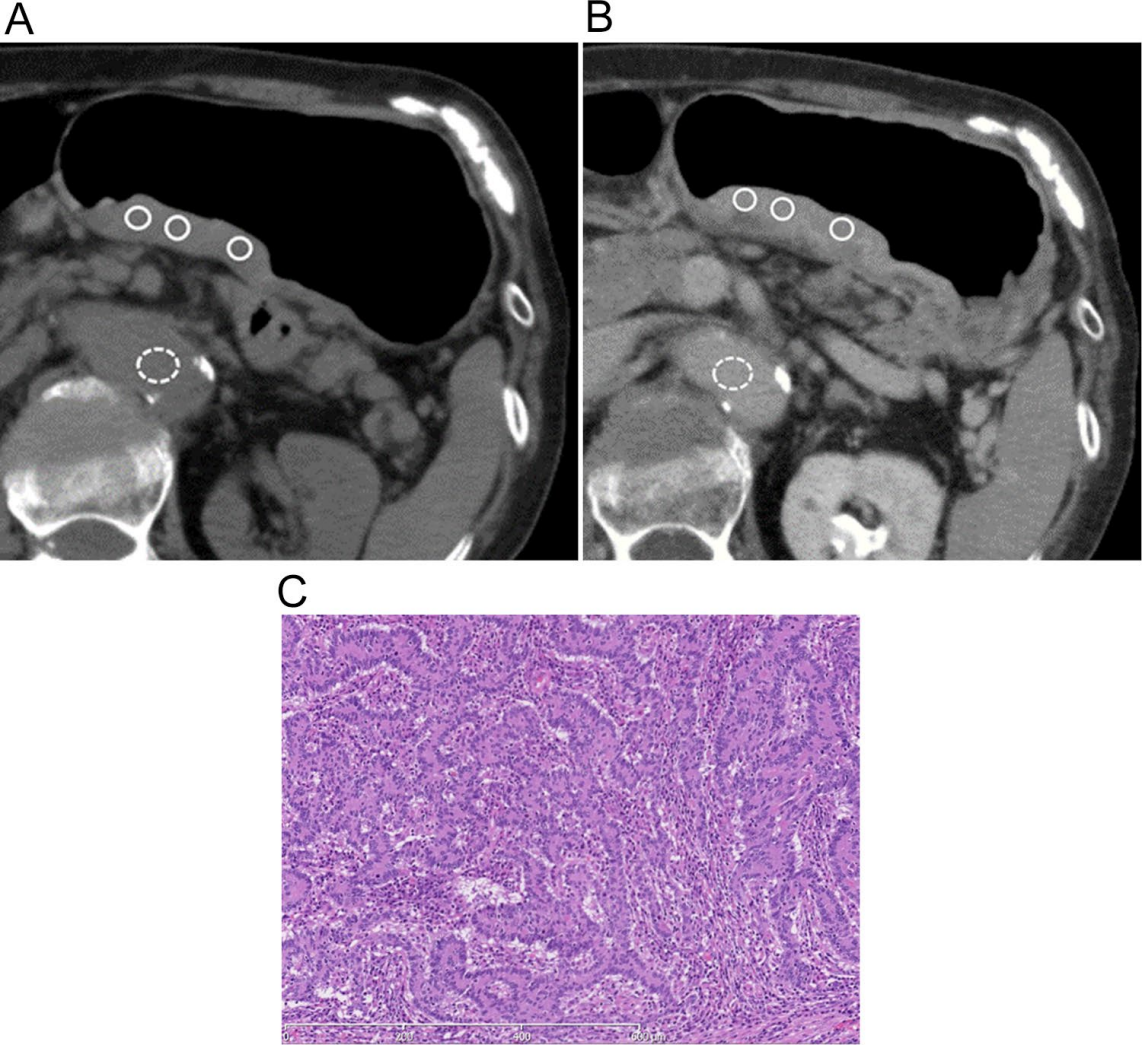
The case of an 82-year-old woman with moderately differentiated adenocarcinoma with solid and expansive proliferation invading the muscularis propria. Axial unenhanced (**a**) and equilibrium-phase contrast-enhanced (**b**) images show the regions of interest on the tumor (white circle) and aorta (white dotted circle). The tumor extracellular volume fraction was 37.5%. A photomicrograph (**c**) shows infiltration of moderately differentiated adenocarcinoma. The tumor cells show a solid and expansive growth pattern (INFa)

**Fig. 2:**
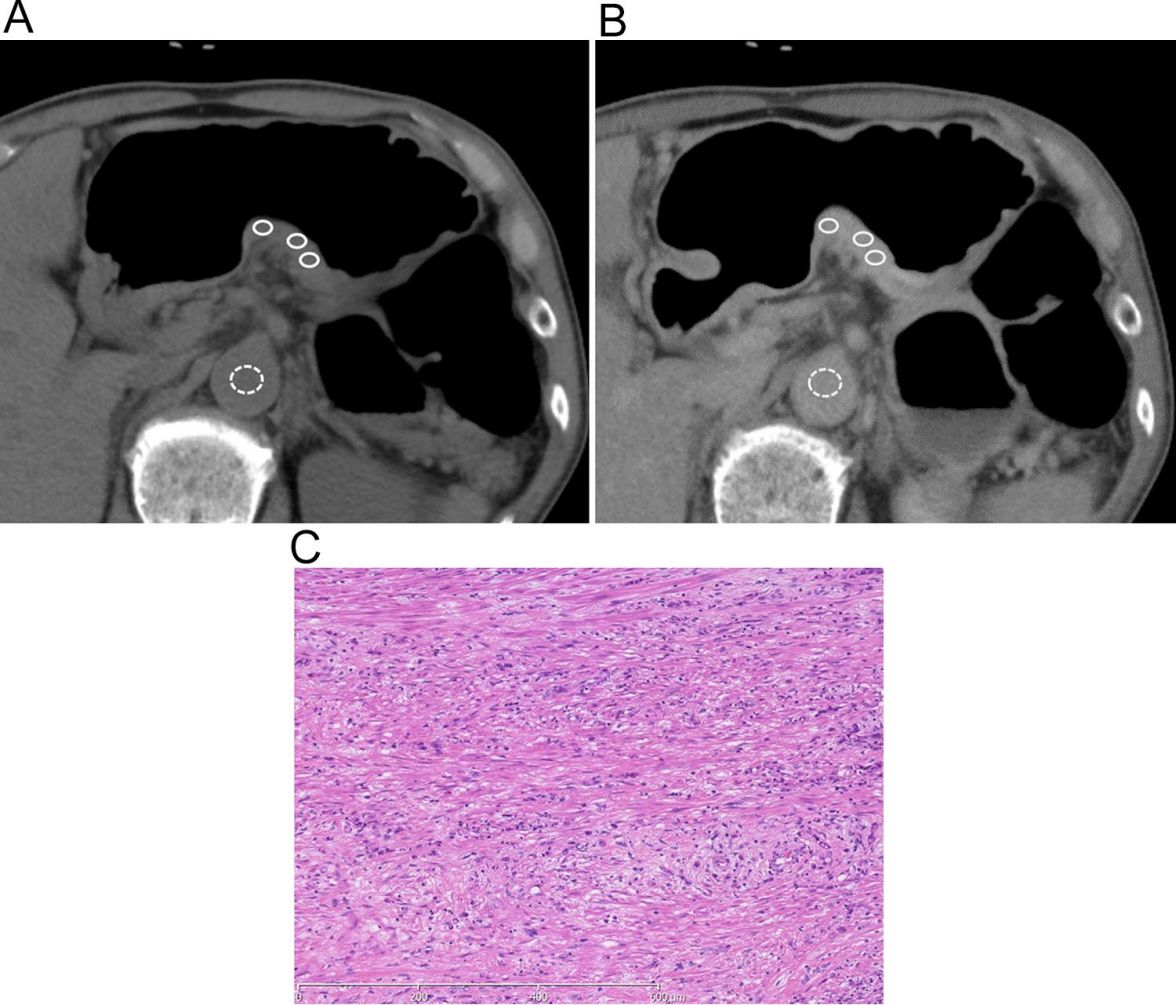
The case of a 72-year-old man with poorly differentiated adenocarcinoma with signet-ring cells and an infiltration growth pattern involving the whole thickness of the gastric wall. Axial unenhanced (**a**) and equilibrium-phase contrast-enhanced CT (**b**) show the regions of interest on the tumor (white circles) in the gastric angle and aorta (white dotted circles). The tumor extracellular volume fraction was 74.9%. A photomicrograph (**c**) shows infiltration of poorly differentiated adenocarcinoma with signet ring cells. The tumor cells show the infiltrative growth pattern with marked fibrosis (INFc)

Attenuation values on unenhanced CT and the equilibrium phase of enhanced CT with 1 mm reconstruction for all GCs were independently obtained by two radiologists (YN and KS, with 14 and 10 years of experience in gastrointestinal radiology, respectively) who did not attend the reading sessions and were blinded to the clinicopathological data; however, the radiologists were aware of the location of the GCs. The readers placed the region of interests (ROIs) within the GC and the aorta at the same level of the GC. Tumor ROIs were positioned in three different areas within the tumor visually representing the greatest enhancement, excluding ulceration, necrosis, and vessel structures. An attempt was made to place the ROIs at identical sites for the unenhanced and equilibrium phase CTs for each patient (Figs. 1, 2). The values measured by the two readers were averaged to represent each ROI. The ECV of each GC was calculated using the following equation: ECV (%) = (1 − hematocrit) × (ΔHUtumor/ΔHUaorta) × 100, respectively, where ΔHUtumor and ΔHUaorta are the HU in the equilibrium phase minus the HU before the contrast agent administration of the tumor and the aorta, respectively [22].

### Pathological findings

Surgical resection was performed according to the Japanese Gastric Cancer Association guidelines [23]. All pathological specimens obtained by gastrectomy were evaluated by experienced pathologists according to the Japanese Classification of Gastric Carcinoma (15th edition) [24]. Tumor location, depth of tumor invasion (T stage), histological type, pattern of tumor infiltration (INF), lymphatic permeation, vascular invasion, and lymph node metastasis were examined and recorded in this study. The histological types were classified as differentiated type (well or moderately differentiated or papillary adenocarcinomas) or undifferentiated type (poorly differentiated adenocarcinoma or signet-ring cell carcinomas). The INF was also classified into three categories: (*i*) INFa, the tumor displays expanding growth with a distinct border from the surrounding tissue; (*ii*) INFb, the tumor shows an intermediate pattern between INFa and INFc; and (*iii*) INFc, the tumor displays infiltrative growth with no distinct border with the surrounding tissue [24].

### Statistical analyses

The statistical analyses were performed with the open-source R platform (Saitama Medical Center, Jichi Medical University, Saitama, Japan) [25]. Differences with *p*-values of < 0.05 were accepted as significant. For univariate analysis, we used Student’s t-test to compare the average of the tumor ECV with each clinicopathological variable. These variables included age (< 65, ≥ 65 years), sex (male, female), CA19-9 (< 37 U/mL, ≥ 37 U/mL), CEA (< 5 ng/mL, ≥ 5 ng/mL), tumor location (upper-, middle- or lower-third), T stage (T1, T2–4), histological type (differentiated, undifferentiated), lymphatic permeation (absent, present), vascular invasion (absent, present), INF (INFa/b, INFc), and lymph node metastasis (absent, present). To identify independent factors that might influence the tumor ECV, we conducted a multiple linear regression analysis to examine the effects of the clinicopathological factors; all variables over *p* < 0.1 in the univariate analysis were included. If significance was indicated, we performed a receiver operating characteristic (ROC) analysis to evaluate the most efficient parameter. Intraclass correlation coefficients (ICCs) were also calculated to evaluate the inter-observer agreement for the ECV: 0.00–0.20, poor correlation; 0.21–0.40, fair correlation; 0.41–0.60, moderate correlation; 0.61–0.80, good correlation; and 0.81–1.00, excellent correlation.

## Results

**Fig. 3:**
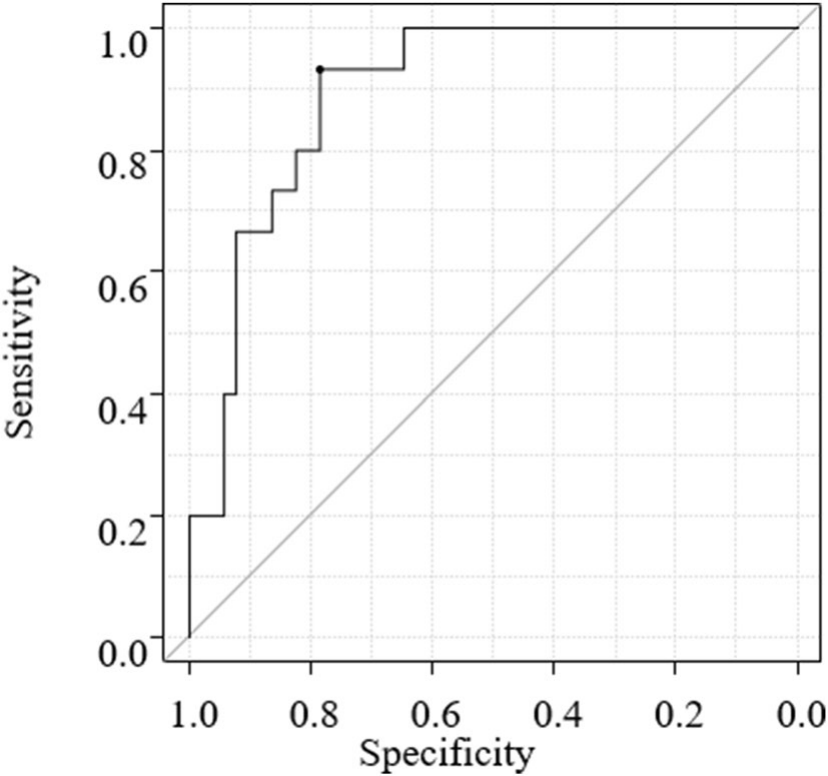
ROC curve of differentiation between the INFc and INFa/p groups. The AUC was 0.89. The optimal cut-off value of ECV to predict the INFc type of gastric cancer was observed to be 60.63%

The characteristics of the patients and tumors are summarized in Table 1. The mean ECV of GC was 54.02% ± 19.42%. In the univariate analysis (Table 2), the mean ECVs were significantly different by venous invasion (absent, 53.6% ± 18.1%; present, 66.1% ± 18.8%; *p* = 0.0487) and INF (INFa/b, 49.7% ± 16%; INFc, 75.1% ± 12.7%; *p* < 0.0001). These results showed that the presence of vascular invasion and INFc were correlated with a higher tumor ECV. We included these two factors as well as T stage (T1, 49.5% ± 18.7%; T2–4, 59.1% ± 17.8%; *p* = 0.069) in the multivariate analysis to further assess their association with the tumor ECV. The results of the multivariate analysis by multiple linear regression revealed significant positive correlations between the ECV and INF only (*β* = 0.54, adjusted *R*^2^ = 0.324, *p* < 0.0001) (Table 3). We obtained an ROC curve comparing the ECV to INF (INFa/b vs INFc). An area under the curve (AUC) value of 0.89 with a 95% confidence interval [95%CI] of 0.817–0.971 was observed, and when the value of 60.63% was used as the cut-off, the sensitivity was 93.3% and the specificity was 78.4% (Fig. 3). The interobserver reproducibility between two readers for measuring the ECV of GC was excellent (ICC = 0.82).

**Table 1:** Characteristics of patients with gastric cancer

Characteristics	Value (*N* = 66)
Age (years; median, range)	65.5 (32–86)
Sex	
Male	33
Female	33
CA 19–9 (U/ml, median, range)	8.15 (0.6–259.6)
CEA (ng/ml, median, range)	1.7 (0.2–13.8)
Tumor location	
Upper-third	14
Middle-third	35
Lower-third	17
T stage	
T1	28
T2	14
T3	11
T4	13
Histological type	
Differentiated	16
Undifferentiated	50
Lymphatic permeation	
Absent	41
Present	25
Vascular invasion	
Absent	56
Present	10
Pattern of tumor infiltration	
INFa	2
INFb	49
INFc	15
Lymph node metastasis	
Absent	39
Present	27

*CA* carbohydrate antigen, *CEA* carcinoembryonic antigen, *T1* invasion of lamina propria or submucosa, *T2* invasion of muscularis propria, *T3* invasion of subserosa, *T4* penetration of serosa or invasion of adjacent structures, *INFa* tumor displays expanding growth with a distinct border from the surrounding tissue, *INFb* tumor shows an intermediate pattern between INFa and INFc, *INFc* tumor displays infiltrative growth with no distinct border with the surrounding tissue

**Table 2:** Univariate analysis of clinicopathological factors

Factor	Number	ECV (% ± SD)	*p*-value
Age			
<65	32	53.3 ± 17.2	0.36
≥65	34	57.5 ± 19.9	
Gender			
Male	33	55.8 ± 17.5	0.897
Female	33	55.2 ± 19.9	
CA19-9			
≤ 37 U/mL	63	55.3 ± 18.8	0.721
> 37 U/mL	3	59.3 ± 14.4	
CEA			
≤ 5 ng/mL	60	55.1 ± 19.1	0.632
> 5 ng/mL	6	59.3 ± 13.1	
Tumor location			
Upper-third	14	52.3 ± 24.0	0.469
Middle- or lower-third	52	56.3 ± 17.0	
T stage			
T1	28	50.64 ± 18.3	0.069
T2–4	38	59.05 ± 18.2	
Histological type			
Differentiated	16	49.6 ± 15.4	0.148
Undifferentiated	50	57.4 ± 19.3	
Lymphatic permeation			
Absent	41	55.6 ± 20.0	0.943
Present	25	55.3 ± 16.3	
Vascular invasion			
Absent	56	53.6 ± 18.1	0.0487
Present	10	66.1 ± 18.8	
Pattern of tumor infiltration			
INFa/b	51	49.7 ± 16.0	< 0.0001
INFc	15	75.1 ± 12.7	
Lymph node metastasis			
Absent	39	52.7 ± 16.8	0.145
Present	27	59.5 ± 20.6	

*CA* carbohydrate antigen, *CEA* carcinoembryonic antigen, *ECV* extracellular volume fraction, *INFa* tumor displays expanding growth with a distinct border from the surrounding tissue, *INFb* tumor shows an intermediate pattern between INFa and INFc, *INFc* tumor displays infiltrative growth with no distinct border with the surrounding tissue, *T1* invasion of lamina propria or submucosa, *T2* invasion of muscularis propria, *T3* invasion of subserosa, *T4* penetration of serosa or invasion of adjacent structures

**Table 3:** Multivariate analysis by multiple linear regression

Variables	Category	*β*	*t*-ratio	*p*-value
T stage	T2–4	−0.04	−0.35	0.73
Vascular invasion	Present	0.16	1.47	0.15
Pattern of tumor infiltration	INF c	0.57	5.09	< 0.0001

*β* standardized regression coefficient

Adjusted *R*^2^ = 0.324

## Discussion

We analyzed the relationship between clinicopathological factors of gastric cancer and the tumor ECV derived from CECT (i.e., the CT-ECV), and the results of our analyses demonstrated that: (*i*) the CT-ECV of the GCs with the INFc pattern was significantly higher than that of the GCs with the INFa/b pattern; and (*ii*) the CT-ECV showed excellent diagnostic accuracy for predicting the pathological INFc pattern.

The pathological INFc pattern is frequently observed in diffuse-type GCs (also known as “scirrhous carcinoma” or “linitis plastica”), and this type of gastric cancer is characterized by diffusely infiltrating lesions that form abundant fibrous stroma as well as poor distensibility of the stomach, depending on the extent of the lesion [26]. The ECV is the sum of the extracellular extravascular space and the intravascular space of a tissue; the former is the site at which fibrosis occurs. Our results may reflect this pathological feature of this type of GC. In this study, the amount of fibrous tumor stroma is not evaluated according to the latest version of the Japanese Gastric Cancer Association guidelines [24]. However, INF is closely correlated with amount of fibrous stroma of GC pathologically [14].

In East Asia, pathological INF has been routinely evaluated as a pathological characteristic of surgically resected hematoxylin and eosin-stained specimens. Several research groups have reported that the pathological INF is closely related to patient prognosis after GC surgery. Saito et al. [6] reported that node-negative patients with an INFc pattern had a poorer prognosis than those with an INFa/b pattern. Nakagawa et al. [27] reported that the pathological INF was closely related to sites of initial recurrence after the curative resection of GC. In their study, patients with the INFc type had a significantly high risk of peritoneal recurrence, whereas those with the INFa/b type had a significantly high risk of hepatic recurrence. Kanda et al. [28] speculated that the INFc pattern may represent a higher penetration ability of cancer cells, which provides a larger probability for peritoneal dissemination. These reports suggest that the INF type may be a biological factor in predicting the prognosis and recurrence site of GC patients after resection. However, the INF is not applicable as a preoperative surrogate marker because it cannot be evaluated using small samples obtained by endoscopic biopsy. Our current results suggest that the CT-ECV may be an alternative in vivo biomarker to the INF of GC. This image-derived biomarker has the advantage of non-invasive evaluation of the entire tumor and excellent reproducibility compared with endoscopic biopsy specimens, which are invasively obtained from a small portion of the tumor.

Several research groups recently described the usefulness of the extracellular extravascular volume fraction (Ve) calculated using dynamic contrast-enhanced MR imaging (DCE-MRI) to evaluate the histological features of GC. Joo et al. [29] reported that the Ve is positively correlated with T staging; the deeper the tumor invasion, the higher the Ve value. DCE-MRI parameters were compared in different histological subtypes of GC by Ma et al. [30]. Their findings revealed that mucinous adenocarcinomas showed higher Ve values compared with non-mucinous tumors, and the diffuse type exhibited higher Ve values than the intestinal type (using the Lauren classification). As these reports showed, the Ve determined by DCE-MRI could reflect the tumor biology and provide histopathological information about GC. However, the use of DCE-MRI may not be commonly applicable as a routine test of GC and may depend on each clinical environment because it is technically demanding and requires multiple repeat studies and complex post-processing steps. The equilibrium CECT approach that we used in our present investigation would be highly versatile in clinical use because its process does not need special systems or equipment. Our method also provides sufficient reproducibility because the inter-observer agreement in the analyses was excellent, indicating that the CT-ECV is a reliable imaging biomarker even in repeatable studies.

Our study has several limitations. It was a single-institution retrospective study with a small sample size. Prospective studies with larger patient numbers would be needed to test our findings. The ROI placement method did not treat heterogeneity of the tumors; only one slice, the largest area of the lesion, was selected for evaluation, and this might have caused sampling errors and image-histopathology mismatching. Moreover, we measured the tumor ECVs using 4-min equilibrium-phase CECT, which might not be a sufficient time for contrast equilibration throughout the tumors. The minimum scan delay for the reliable estimation of the ECV has not been well standardized. Several authors calculated the ECV fraction of other organs at the equilibrium phase for 10 min or longer after the initiation of contrast materials [17, 18]. Further studies are needed to address these issues.

In conclusion, the ECV determined by equilibrium CECT could serve as a promising and non-invasive index to predict the infiltration growth pattern of GCs, which might optimize treatment strategies for patients. The CT-ECV could potentially become a reliable imaging biomarker for GC.
